# *Toxoplasma gondii* infection triggers chronic cachexia and sustained commensal dysbiosis in mice

**DOI:** 10.1371/journal.pone.0204895

**Published:** 2018-10-31

**Authors:** Jessica A. Hatter, Yue Moi Kouche, Stephanie J. Melchor, Katherine Ng, Donna M. Bouley, John C. Boothroyd, Sarah E. Ewald

**Affiliations:** 1 Department of Microbiology, Immunology and Cancer Biology and the Carter Immunology Center, University of Virginia School of Medicine, Charlottesville, VA, United States of America; 2 Department of Comparative Medicine, Stanford University, Stanford CA, United States of America; 3 Department of Microbiology and Immunology, Stanford University, Stanford CA, United States of America; Centre National de la Recherche Scientifique, FRANCE

## Abstract

*Toxoplasma gondii* is a protozoan parasite with a predation-mediated transmission cycle between rodents and felines. Intermediate hosts acquire *Toxoplasma* by eating parasite cysts which invade the small intestine, disseminate systemically and finally establish host life-long chronic infection in brain and muscles. Here we show that *Toxoplasma* infection can trigger a severe form of sustained cachexia: a disease of progressive lean weight loss that is a causal predictor of mortality in cancer, chronic disease and many infections. *Toxoplasma* cachexia is characterized by acute anorexia, systemic inflammation and loss of 20% body mass. Although mice recover from symptoms of peak sickness, they fail to regain muscle mass or visceral adipose depots. We asked whether the damage to the intestinal microenvironment observed at acute time points was sustained in chronic infection and could thereby play a role in sustaining cachexia. We found that parasites replicate in the same region of the distal jejunum/proximal ileum throughout acute infection, inducing the development of secondary lymphoid structures and severe, regional inflammation. Small intestine pathology was resolved by 5 weeks post-infection. However, changes in the commensal populations, notably an outgrowth of *Clostridia spp*., were sustained in chronic infection. Importantly, uninfected animals co-housed with infected mice display similar changes in commensal microflora but never display symptoms of cachexia, indicating that altered commensals are not sufficient to explain the cachexia phenotype alone. These studies indicate that *Toxoplasma* infection is a novel and robust model to study the immune-metabolic interactions that contribute to chronic cachexia development, pathology and potential reversal.

## Introduction

Chronic diseases account for over 85% of deaths in the first world and 70% of deaths globally[1]. The co-occurrence of cachexia, or the progressive loss of lean body mass, is one of the best predictors of mortality across chronic disease. Cachexia is distinct from starvation or malabsorption and can be accompanied by anorexia, elevated inflammatory cytokines (IL-1, IL-6 and TNF-α), loss of fat and insulin resistance[2]. In human disease, therapeutic interventions including nutritional supplementation, appetite stimulants, steroid treatment and TNF-α inhibitors have not proven widely successful to block or reverse cachexia[3]. This may be linked to limitation of current animal models of cachexia which fail to recapitulate the chronic nature of clinical disease. Specifically, low-dose endotoxin injection causes weight loss over a period of several days, but after repeated injections, mice develop tolerance and return to normal weights. Cancer cachexia models may take many weeks before the onset of symptoms, however, the period of weight loss is also limited to 1–2 weeks before animals succumb to the tumor. Renal and cardiac obstruction models have a similar 1–2 week time frame of disease before animals succumb to the surgery[4]. Thus, there is a great need to develop animal models that recapitulate the long-term nature of clinical cachexia both to understand the underlying mechanisms of disease and to test targets for disease intervention and potential reversal.

*Toxoplasma gondii* is an obligate intracellular protozoan parasite that cycles between a broad range of mammalian intermediate hosts and definitive feline hosts. Intermediate hosts are infected for life and support haploid division/asexual expansion of parasite strains. Intermediate hosts are infected when they ingest either oocysts shed in cat in feces or tissue cysts, termed bradyzoites, in the muscle or brain of other intermediate hosts. Over the first three days post-ingestion, *Toxoplasma* migrates down the small intestine, converts to the rapidly dividing tachyzoite stage, and infects intestinal epithelial cells and immune cells[5–7]. Acute infection is marked by severe, focal disruption of the villi, expansion of secondary lymphoid structures and the appearance of “casts” formed from matrix and dead cells that form a physical barrier over damaged regions of the ileum[7]. Several groups have reported a decrease in microbial diversity in the gut, marked by outgrowth of Gram negative bacterial species, as well as commensal microbe translocation to the liver[7–9]. However, whether these alterations to commensal homeostasis are maintained during chronic infection has not been asked.

*Toxoplasma* benefits from local intestinal inflammation by infecting infiltrating monocytes and dendritic cells and using them to traffic throughout the host[10]. Over the course of 3–4 weeks, a Th1-mediated adaptive immune response clears systemic parasitemia, except in select tissues (mainly the brain and skeletal muscle) which support stage conversion to bradyzoite tissue cysts. The bradyzoite form *of Toxoplasma* is characterized by altered transcriptional profiles, a shift to glycolytic metabolism, slow growth, and formation of a polysaccharide-rich wall that protects the parasites as they transit through the stomach of the subsequent host[11]. Thus, parasite transmission requires a robust host immune response; ensuring that the host survives acute infection and enabling the parasite to access the tissues amenable for chronic infection. Once the parasite has converted to the bradyzoite form, transmission requires predation of the chronically infected host. Cats acquire *Toxoplasma* by eating intermediate hosts and play an important role in the parasite life cycle by: 1) facilitating sexual recombination of the parasite, thereby increasing genetic diversity; and 2) mediating range expansion of the parasite by shedding millions of highly stable and highly infectious oocysts[12,13]. The selective advantage conferred by infecting cats and the predator-prey relationship between cats and rodents suggest that mice and rats are critical intermediate hosts for *Toxoplasma*. The importance of this relationship is evident in the sophisticated mechanisms the parasite has evolved to intersect host signaling pathways[14], promoting intracellular survival; as well as the observation that *Toxoplasma* infected rodents lose their aversion to cat urine, a putative means to facilitate transmission[15,16].

Here we show that in the first 10 days post-*Toxoplasma* infection adult mice lose 20% of their body mass, which is associated with elevated circulating cytokines, anorexia and moribund behavior. The majority of *Toxoplasma* infected animals do not succumb to infection yet the reduction of muscle mass and visceral white adipose depots is sustained, indicating that *Toxoplasma* infection is a robust and reproducible model of sustained cachexia. We show that *Toxoplasma* infects and replicates in distinct puncta along the distal jejunum and proximal ileum throughout the acute phase of infection. Peak inflammation correlates directly with parasite load but is resolved by 5 weeks post-infection. Using 16S sequencing, we identify an outgrowth of *Clostridia spp*. that is sustained during the chronic stages of disease. Importantly, co-housed uninfected animals exhibit a similar shift in commensal populations without exhibiting any signs of illness or weight loss, consistent with the conclusion that commensal alterations alone are not sufficient to explain the sustained cachexia disease. We propose that promoting muscle and fat wasting may be a means of enhancing the opportunity for rodent predation and transmission of this parasite to definitive feline hosts.

## Materials and methods

### Animals

CBA/J, BALB/cJ and C57BL/6J mice were purchased from Jackson Laboratories. Animals were housed in BSLII level conditions. All animal protocols were approved by Stanford University's Administrative Panel on Laboratory Animal Care (Animal Welfare Assurance # A3213-01, protocol # 9478) or the University of Virginia Institutional Animal Care and Use Committee (protocol # 4107-12-15). All animals were housed and treated in accordance with AAALAC and IACUC guidelines at the Stanford School of Medicine or the University of Virginia Veterinary Service Center.

### Parasites, cells and cell lines

The parasite strain used for these studies was Me49 that stably expresses green fluorescent protein and luciferase, and has been previously described (17). Parasites were passaged intracellularly in human foreskin fibroblasts (ATCC) and passaged by 25G syringe lysis in complete DMEM (cDMEM, Gibco) plus 10% FBS (HiClone), 100ug Penicillin-Streptomycin (Gibco) and 1mM Sodium Pyruvate (Gibco).

### Infections

To generate cysts, 6–8 week-old female CBA/J mice were infected with 1000 Me49 tachyzoites stably expressing green fluorescent protein and luciferase (Me49-gfp-luc) by intraperitoneal injection. 4–8 weeks following infection, mice were euthanized with CO_2_ and brains were harvested, homogenized through a 50 μm filter, washed 3 times in PBS, stained with dolichos biflorus agglutinin-rhodamine (Vector labs) and the number of cysts were determined by counting rhodamine GFP double-positive cysts at 20x magnification. Prior to infection, 8–10 week male mice were cross-housed on dirty bedding for two weeks to normalize commensal microbiota. Mice were fasted overnight and fed between 100 and 250 Me49-GFP-luc cysts on ¼ piece of mouse chow in individual cages. Weights and health were monitored daily. This method was chosen over gavage based on the data from Boyle et al. 2007 showing that gavage can cause epithelial damage and parasite invasion in the oral cavity or esophagus, disrupting normal patterns of dissemination[17].

### Assessment of food intake

To measure food intake, mice were house on chip bedding and food was weighed daily when added to the cage. When animals began showing signs of sickness (1 week post infection) several pieces of food were place in dishes on the floor of the cage which encouraged animals to eat without having to reach for the hopper. After 24 hours the food was removed from the hopper, the bedding was sifted to find intact pieces of food, and all remaining food was weighed. The 24-hour difference in food weight was normalized to the total body weight of all mice in the cage to determine the amount of food eaten. Of note, interventions including addition of moistened food, high caloric supplements or intra peritoneal injection of fluids were not implemented in this study. Extra nesting supplies were maintained in cages for the duration of the study.

### 5-point sickness score to assess likelihood of recovery

Mice were monitored daily for 1) ruffled fur, 2) hunching, 3) >20% weight loss, 4) squinting/eye discharge, 5) failure to move on an open hand. Mice that scored 5/5 based on these parameters were immediately euthanized. In chronic cachexia, most animals retain a sickness score of 1–2 due to weight loss and/or fur ruffling. Roughly 1/100 mice would be found dead in a cage, however, as this often occurred after the resolution of peak sickness, it is likely that these deaths were due to invasion of the parasite in a critical region of the brain leading to rapid lethality unrelated to other aspects of disease.

### Tissue harvesting and cytokine measures:

At the experimental end points, mice were euthanized with CO2. Blood was isolated by cardiac exsanguination. Abdominal subcutaneous white adipose depots, epididymal visceral white adipose depots, supraclavicular brown adipose depots, quadriceps, tibialis anterior, EDL and gastrocnemius muscles were isolated and placed in pre-weighed 2mL tubes for weighing and flash freezing. For small intestine, a Peyer’s Patch containing regions of the distal jejunum or ileum were identified by eye. A 2cm section surrounding the Peyer’s patch (or patches) was excised. Sections immediately adjacent to but excluding a Peyer’s patch were harvested as well. Sera cytokines levels were measured by Luminex cytokine array at the Stanford Human Immune Monitoring Core or at the University of Virginia Flow Cytometery Core.

### Bioluminescence imaging and quantification

For bioluminescence imaging (BLI), mice were injected in the intraperitoneal cavity with 200 μL of a 15 mg/mL stock solution of luciferin (Xenogen), anesthetized with isoflurane and imaged for 4 minutes on an IVIS system. To image organs, mice were injected 5 minutes prior to euthanasia, after which the organs were harvested and imaged for 4 minutes. Images were analyzed with LivingImage software and ImageJ.

### Histology and microscopy

At the experimental end points, mice were euthanized with CO_2_, after which tissues were harvested and subjected to IVIS imaging and fixation in formalin. Samples were submitted to the Stanford Department of Comparative Medicine Histology Core for paraffin embedding and sectioning. Regions of the jejunum were selected based on the presence of a Peyer’s patch. Adjacent regions of the Jejunum (not containing a Peyer’s patch) were also harvested. Every other section was stained with H&E. A semi quantitative scoring system of 1 to 5, (1 = no significant lesion, 2 = mild, 3 = moderate, 4 = marked, 5 = severe) was used to evaluate the severity of any lesions. Parameters included inflammatory cellular infiltrate, loss of Peyer’s patch organization, villi destruction and villi shortening. For detailed scoring, each tissue section was blinded, divided into fields of view at 40x and an inflammation score was assigned to each field of view.

Unstained sections were used to quantify *Toxoplasma* load. To deparaffinize, sections were passed twice through xylene, then through 100% ethanol, 80% ethanol and 50% ethanol and distilled water for 3 minutes each. Antigen retrieval was performed by incubating sections in sodium citrate buffer brought to a boil in the microwave and incubated for 15 minutes in a vegetable steamer (10mM Citric Acid, 0.05% Tween-20, pH6.0). Slides were cooled, washed once in PBS and outlined with a Pap pen to perform staining. Samples were blocked for 30 minutes in 5% goat sera in PBS. *Toxoplasma* was labeled with mouse anti-*Toxoplasma*-FITC (Thermo Scientific Clone J26A) at a concentration of 1 μg/μL in 5% goat sera overnight. Samples were washed 3x in PBS, mounted in Vectashield with DAPI (Vector Laboratories) and imaged on an Olympus BX60 upright fluorescence microscope with a 4x, 10x, 40x or 100x objective. To quantify parasite load, each section was subdivided identically to the adjacent H&E section. The threshold of parasite signal at 488nm was determined by comparison to uninfected samples, each image was converted to binary and the dark pixels were counted using ImageJ.

### 16S ribosomal sequencing and diversity analysis

Fresh fecal pellets were collected from mice at the time points indicated and flash frozen. DNA was isolated using the MoBio PowerSoil Kit and barcoded primers were used to amplify the V4 region of the 16S rRNA gene. MoBio UltraCLean-htp 96 Well PCR Clean-Up Kit was used to purify PCR products which were then quantified using Quant-iT ds DNA Assay Kit. 184 samples were pooled at equimolar ratios. 16S ribosomal sequencing was performed by the Mayo Clinic using a single lane of the Illumina HiSeq. Community composition and beta diversity were determined using QIIME and beta diversity was visualized using EMPeror[18,19]. T-tests were performed using GraphPad Prism and corrected for multiple hypothesis using the FDR approach.

### qPCR

Flash frozen Peyer’s patches were homogenized and RNA was isolated using Trizol. RNA. For each sample 10μg of RNA was digested and converted to cDNA using the qMax cDNA Synthesis Kit (Accuris). 7.2μL of cDNA was used in each 20μL qPCR reaction. *Toxoplasma* cDNA was detected using forward primer (5′-CACAGAAGGGACAGAAGT CGAA-3’), reverse primer (5’-CAGTCCTGATATCTCTCCTCCAAG A-3’), and a probe (FAM-5′-CTACAGACGCGATGCC-3′)[20]. Beta actin levels were determined using a Taqman probe Mm02619580_g1 (ThermoFisher). qPCR was performed on a QuantStudio6 instrument (Applied Biosystems)

## Results and discussion

**Fig 1:**
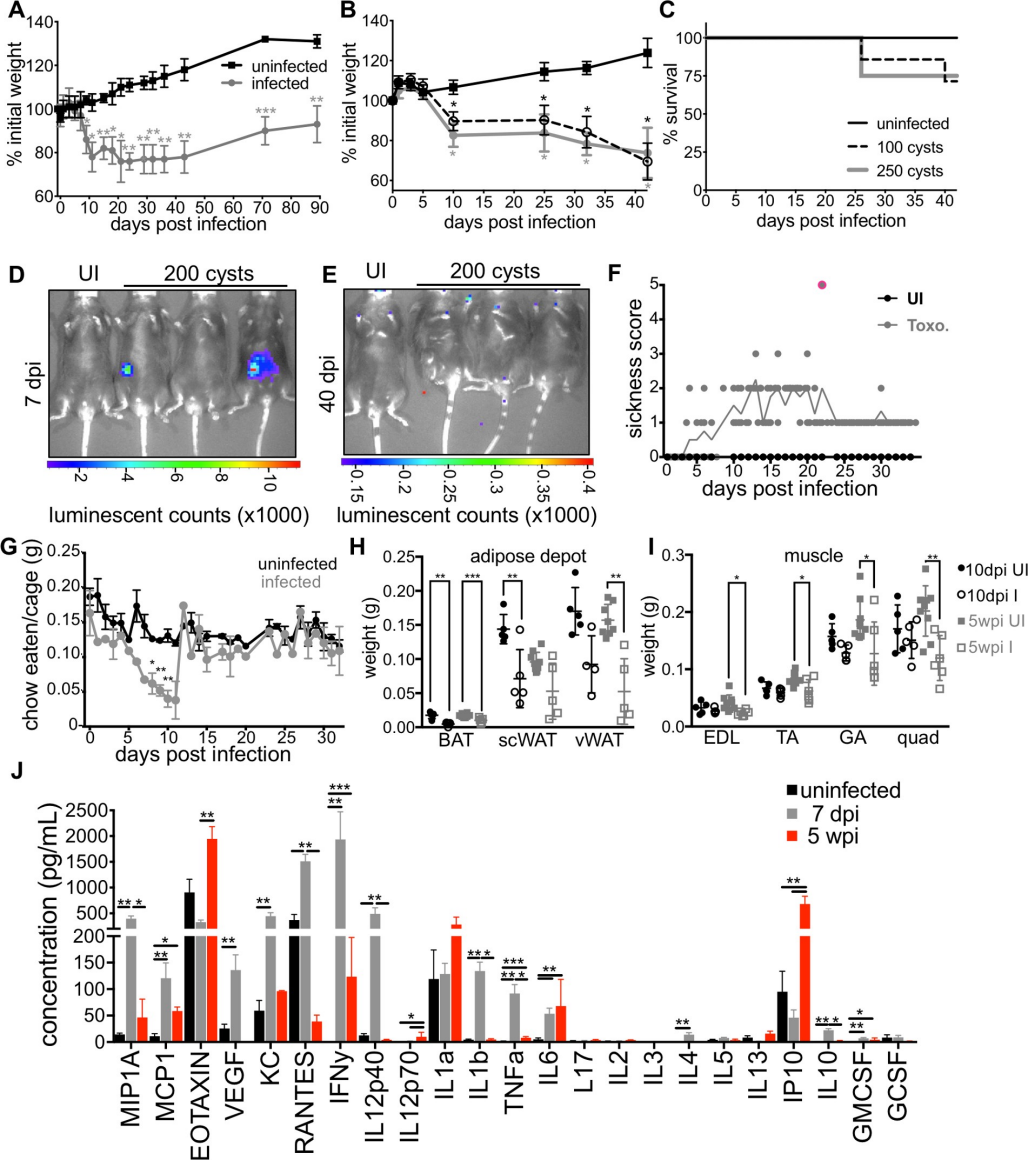
C57BL/6 mice infected with *Toxoplasma* become chronically cachexic. **A**, Following per oral infection with 120 Me49-GFP-luciferase cysts (grey) or mock infection (black) mice were monitored for weight loss. Data average of 8 experiments. **B**, Mice were infected as described in (**A**) with 100 cysts (dashed line), 250 cysts (grey) or mock infected (black) weight was monitored at the indicated time points. **C**, Survival curves for mice represented in (**B**). N = 4–7 mice per group, data representative at least 3 experiments. **D**, Mice were harvested at 7 days post infection (dpi) (**E**) or 40 dpi to assess parasite load by bioluminescence assay. **F**, Sickness scores for animals infected per orally with 100 Me49gfp-luc cysts (grey circles, toxo) or mock infected with PBS (black circles, UI). Animals were scored on a scale of 1–5: hunched posture, ruffled fur, failure to move when picked up, eyes closed or discharged, loss of over 20% weight. Mice were monitored twice daily at peak sickness and euthanized immediately if all 5 behaviors were observed (red outline). N = 5 mice per group. **G**, Every 24 hours food was weighed to determine the amount eaten and normalized to the weight of animals in the cage. Data are average of 2 experiments, N = 5 mice per group each experiment. Significance relative to uninfected at the same time point. **H**, Brown adipose tissue (BAT), sub cutaneous white adipose tissue (scWAT) or visceral white adipose tissue (vWAT) was harvest at 10 dpi (black) or 5 wpi (grey) and weighed. **I**, Extensor digitorum longus (EDL), tibialis anterior (TA), gastrocnemius (GA) and quadriceps (QUAD) muscles were weighed at 10 dpi (black) or 5 wpi (grey) post infection. Data is average of 2 experiments, N = 5–10 mice per time point. **J**, Luminex cytokine array was performed on sera from uninfected mice (black), 7 dpi (grey) or 5 wpi (red) mice. N = 3–10 mice per group. * p≤0.01, ** p≤0.001, ***p≤0.001, SEM, student’s T-test.

**Fig 2:**
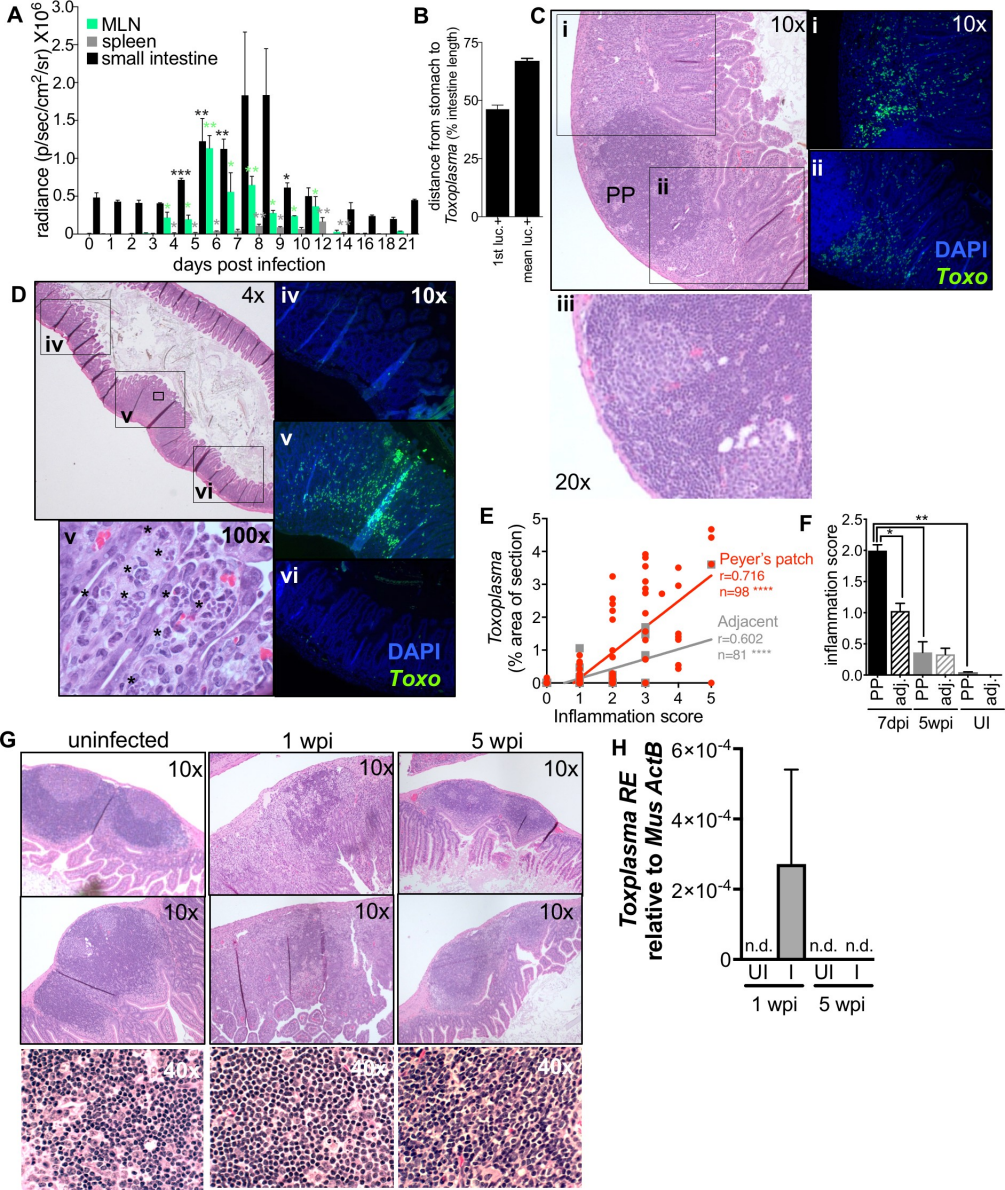
Mice recover from severe acute inflammation and parasite growth in the small intestine. **A-B**, Following per oral infection with 200 Me49-GFP-luc cysts, mice were euthanized and parasite load was determined by BLI in the small intestine (black), mesenteric lymph nodes (MLN, green) and spleen (grey). N = 3 animals per day, Significance is measured for each time point relative to day 3 post infection. Student’s T-test * p≤0.01, ** p≤0.001. **B**, For each intestine, the position of the luciferase signal was measured as distance from the stomach to the first luciferase positive region (1^st^ luc+) or distance from the stomach to the mean of all luciferase positive regions (mean luc +). The position of luciferase signal was averaged from 4 to 10 dpi. **C**-**H**, 2cm segments of the distal jejunum/ileum (the distal 50–90% of the small intestine) were excised for histology based on the presence of Peyer’s patches visible to the eye at 7dpi, 5wpi or from uninfected mice. **C**-**D**, Representative images of 7dpi sections stained with H&E to assess inflammations score at 10x or 20x (**iii**) magnification (scale of 1 = no detectable inflammation to 5 = complete disruption of lymphoid structure and/or villi). Parasites vacuoles were visible in the villi at 100x magnification (**v**, asterisks). In addition to staining a section for H&E, an adjacent section of each intestinal segment was stained with a *Toxoplasma*-specific antibody (green) and DAPI (blue) and imaged at 10x (**i**-**vi**) to assess parasite load. **E**, Correlation between parasite load and inflammation score in intestinal segments 7dpi. Segments containing a Peyer’s patch (red) or adjacent segments lacking a Peyer’s patch (grey), Pearson’s correlation **** p≤0.0001. Slopes were significantly different between the two groups: Peyer’s patch 0.781±0.078, adjacent 0.296±0.044 p<0.0001, linear regression of correlations. **F**, Inflammation score for Peyer’s patch-containing segments (solid bars) and adjacent, Peyer’s patch negative segments (dashed bars) of the intestine from uninfected animals, 7 dpi (black) or 5wpi (grey). Student’s T-test *p≤0.05, ** p≤0.001, SEM, N = 4–6 segments from 3 mice per condition. **G**, Representative images of Peyer’s patch organization in uninfected, 1wpi and 5 wpi small intestines. **H,** Parasite load in the Peyer’s patch containing intestine segments 1wpi or 5wpi using quantitative PCR. *Toxoplasma R*E expression relative to host *actB*. N = 5.

10–12 week male C57BL6 mice acquired from Jackson Labs were cross-housed on dirty bedding for two weeks. Mice were then infected per orally with 100–250 *Toxoplasma* cysts of the Me49 background engineered to express GFP and luciferase (Me49-GFP-luc). Body weight was monitored for the duration of infection. Mice lost a significant amount of weight during the acute phase of infection (7 to 14 days post infection (dpi), but weight loss stabilized by 30 dpi, the onset of chronic infection (Fig 1A). Although mice increased in weight over the chronic phase of infection (day 30–90) they remained 20% less massive than uninfected controls. Animals infected with 100 or 250 cysts had similar weight loss (Fig 1B) and survival through 40 dpi (Fig 1C). Although the precise reason for similar disease kinetics across this range of cyst doses is not known, these data suggest a bottleneck at the intestine that prevents parasites from disseminating systemically beyond a certain threshold. Parasites were sometimes visible by bioluminescence assay when imaged ventrally at day 7 dpi (Fig 1D); however, by day 40 dpi, parasite signal was not detectable (Fig 1E). Of note, we have determined that luminescence signal can be masked if the parasites invade a region of the intestine on the dorsal face of the abdominal cavity. Despite variability in ventral luciferase signal, infections were very similar across small intestines when imaged ex vivo (Fig 2).

Because infected mice routinely lose over 20% of their initial body weight but recover other indicators of health, we determined that weight loss alone was not an accurate indicator of morbidity. We implemented a 5-point sickness score (ruffled fur, hunching, >20% weight loss, squinting or eye discharge, failure to move) to differentiate between animals were almost certain to recover versus animals that required euthanasia (Fig 1F). Most animals routinely exhibit 3/5 phenotypes or above for a few days. Any animals scoring 5/5 were immediately euthanized (Fig 1F red). During chronic infection most animals retained a score of 1–2 based on reduced body mass and fur ruffling. Although mice underwent a phase of anorexia during acute infection, they regained their appetites and consumed normal pre-infection food amounts by 15dpi indicating that sustained weight loss was not simply due to sustained anorexia (Fig 1G). These results are consistent with a 1997 report from Arsenijevic et al. that showed that mice infected with *Toxoplasma* can be described in different response groups: death in acute infection, failure to regain body mass or partial recovery of body mass[21]. However, the major tissues affected were not determined.

To identify the tissue types affected, abdominal subcutaneous fat depots (scWAT, a rapidly mobilized energy source), epididymal visceral white adipose depots (vWAT, a key metabolic regulatory tissue), and supraclavicular brown adipose depot (BAT, thermogenic fat) were harvested. At 10 dpi, there was already significant reduction in BAT and scWAT depots. VWAT depots were significantly reduced 5 weeks post infection (wpi) (Fig 1H). In contrast to fat depots, which were reduced early, tibialis anterior (TA), gastrocnemius (GA) and quadriceps (QUAD) muscles were significantly reduced at 5wpi (Fig 1I). This sustained muscle loss was consistent with a recent report describing T regulatory cell-dependent myositis during chronic *Toxoplasma* infection which leads to impaired animal strength[22].

At 7 dpi, the canonical cachexia cytokines IFN-γ, IL-1β, TNF-α and IL-6 were significantly upregulated in the sera (Fig 1J, grey versus black). IFN-γ, specifically, is well described to be necessary to control chronic parasite infection in mice[23–26]. Consistent their potent inflammatory capacity, at chronic infection serum IFN-γ ad TNF-α were substantially reduced compared to acute infection. However levels of IFN-γ, TNF-α and IL-6 remained significant compared to uninfected mice (Fig 1J, red versus black). Among the significantly upregulated in chronic infection were eotaxin, a CCR3 agonist (a receptor highly expressed on TH2 cells), and IP10, which has been reported to antagonize the same receptor, suggesting a complex layering of pro and anti-inflammatory signals in chronic infection[27]. Elevated sera titers of eotaxin, IP-10 and MCP-1 (a chemoattractant for macrophage and T cells) have also been observed in long-term follow up on a cohort of patients with juvenile dermatomyositis, a disease characterized by inflammatory cells surrounding the blood vessels leading to destruction of muscle fibers and skin irritation[28]. IP-10 upregulation is consistent with the sustained elevation in IFN-γ[23,29,30]. Cumulatively these data indicate that chronic infection with *Toxoplasma* meet a modern definition of cachexia put forth in 2008: the loss of 5% or more lean body mass accompanied by anorexia, fat loss and inflammation (IL-1, TNF, IL-6, acute phase proteins) and/or insulin resistance[2].

Previous studies have suggested that C57BL/6 mice are uniquely susceptible to infection with Me49 parasites[31]. Importantly, the cachexia phenotype was not restricted to C57BL/6 mice as CBA/J mice also lost approximately 20% of their body mass (S1 Fig, black vs. blue solid lines). To show that cachexia following *Toxoplasma* infection was not simply an artifact of our infection protocol or animal house conditions we infected BALB/c mice. Whereas C57BL/6 and CBA/J mice express the H-2D^b^ haplotype of MHC class I, BALB/c mice express the H-2L^d^ haplotype of MHC class I[32]. BALB/c H-2L^d^ presents the *Toxoplasma* dense granule protein GRA6, leading to a more effective CD8-mediated immune response, limited acute parasitemia, decreased chronic cyst burden, decreased overall inflammation and minimal if any weight loss compared to C57BL/6[33]. As expected, BALB/c mice were protected from all weight loss using our experimental conditions (S1 Fig, black vs orange solid lines).

*Toxoplasma* is naturally acquired by ingestion of oocysts or tissue cysts leading to severe regional inflammation in the small intestine[7,9,34]. We reasoned that cachexia could be the result of sustained gut inflammation, changes in intestinal architecture and/or the gut commensal community. To address this question, we orally infected mice with 200 Me49-GFP-luc cysts. Three mice per day were euthanized to assess parasite load in the small intestine, mesenteric lymph node and spleen by bioluminescence assay. Significant parasite signal was observed in the small intestine at 4 dpi and peaked 7–8 dpi (Fig 2A, black bars). Parasites were observed in the mesenteric lymph nodes (Fig 2A, green bars) and spleen (Fig 2A, grey bars) as early as day 3. For as long as *Toxoplasma* was detected by BLI (Fig 2A, 4-10dpi) the first luciferase signal was consistently found at 50% the length of the small intestine and the mean of all luciferase positive regions was identified at 2/3^rd^ the length of the intestine (Fig 2B) indicating that the parasites grow primarily in this niche for the majority of the time they are found in the small intestine.

These data are consistent with previous reports studying the first week of infection. Specifically, Gregg *et al*. have shown parasite infection along the mucosa of the small intestine in the duodenum, jejunum and ileum over the first 6 days of infection[6]. Further, Molloy *et al*. demonstrated that 9 dpi, commensals were segregated from the epithelial layer in the ileum but not the jejunum by the presence of a ‘cast’-like pseudomembrane composed of dead host cells and invasive *E*. *coli* suggesting that there is a distinct interplay between *Toxoplasma*, commensals and the immune system in this tissue[7]. While we did not observe parasite signal in the duodenum, this may be due the fact that we imaged intestines from the serosal side rather than the luminal aspect. In addition, the bioluminescence assay is not sensitive enough to detect small numbers of parasites that may be present elsewhere in the small intestine[6]. Nonetheless, our data are consistent with the interpretation that the distal jejunum/proximal ileum is the major small intestinal niche for parasite replication throughout acute infection. This region of the small intestine is enriched in immune resident cells, specialized structures including M cells that allow for sampling of the lumen and can mediate pathogen transit into the host, as well as an expansion in microbial diversity. All elements could contribute to *Toxoplasma’s* predilection for residence in this niche[35].

Diet as well as reactive oxygen species derived from inflammatory infiltrate can produce auto-luminescent signal. To validate that the luciferase signal was derived from *Toxoplasma*, and to monitor the degree of inflammation, we harvested segments of the small intestine 7 dpi for histological analysis. Having observed that luciferase positive regions always occurred adjacent to at least one enlarged Peyer’s patch, 2cm segments centered on a Peyer’s patch (or Peyer’s patches) were excised from the small intestine of infected and uninfected mice (Fig 2C–2G). This allowed us to assess parasite load and the degree of inflammation in matched regions of the intestine across time points without pre-existing knowledge about parasite location provided by BLI. These intestine segments were fixed and sectioned. One section was stained with H&E (Fig 2C, 2D and 2G) to assess inflammation. The adjacent section was de-paraffinized and stained for *Toxoplasma* using an antibody specific to parasite lysate and for nuclei using DAPI (Fig 2i-vi). At 7 dpi, tachyzoites were observed throughout the villi and the lamina propria. Interestingly, in sections where a Peyer’s patch was cross-sectioned, parasites were observed nearby but excluded from lymphoid follicle (Fig 2Ci and 2Cii). Necrosis was not observed in Peyer’s patches at 7dpi (Fig 2Ciii 20x and 2G 40x). When H&E sections were examined at high magnification, vacuoles containing multiple tachyzoites were visible in intestinal epithelial cells, indicating that parasites were growing in this niche at 7dpi (Fig 2Dv, 100x, asterisks).

We noticed the fields of view closest to the Peyer’s patch contained the most *Toxoplasma* (Fig 2D v versus iv and vi fluorescence images 10x), whereas neighboring fields of view contained few parasites and were less morphologically disrupted (Fig 2D iv and vi 2H & 2E). To quantify this observation, 2cm segments of the intestine containing a Peyer’s patch or 2cm segments immediately adjacent to but excluding Peyer’s patches were isolated, sectioned, and stained for H&E or *Toxoplasma* as described above. Across each section, there was a significant positive correlation between inflammation score and parasite load (Fig 2E Pearson’s correlation, Peyer’s patch segments, red: r = 0.716, n = 98, p<0.0001; adjacent segments, grey: r = 0.602, n = 81, p<0.0001). Peyer’s patch negative sections had a significantly lower overall inflammation score and parasite load (Fig 2E linear regression of correlations, Peyer’s patch segments: 0.781±0.078; Adjacent segments: 0.296±0.044, p<0.0001 and Fig 2F). By 5 wpi there was not a significant difference in inflammation score between infected and uninfected intestinal segments (Fig 2F). Also consistent with the conclusion that infection in the small intestine was resolved in chronic disease, Peyer’s patch architecture, which had a highly disorganized germinal center 7 dpi, was indistinguishable from uninfected animals by H&E staining 5wpi (Fig 2G). Finally, *Toxoplasma* was no longer observed in the Peyer’s patch-containing segments of the small intestine by 5 weeks post infection by Q PCR (Fig 2H). Taken together, these results indicate that acute inflammation in the small intestine is resolved by chronic infection and is therefore unlikely to drive the sustained cachexia in these animals.

**Fig 3:**
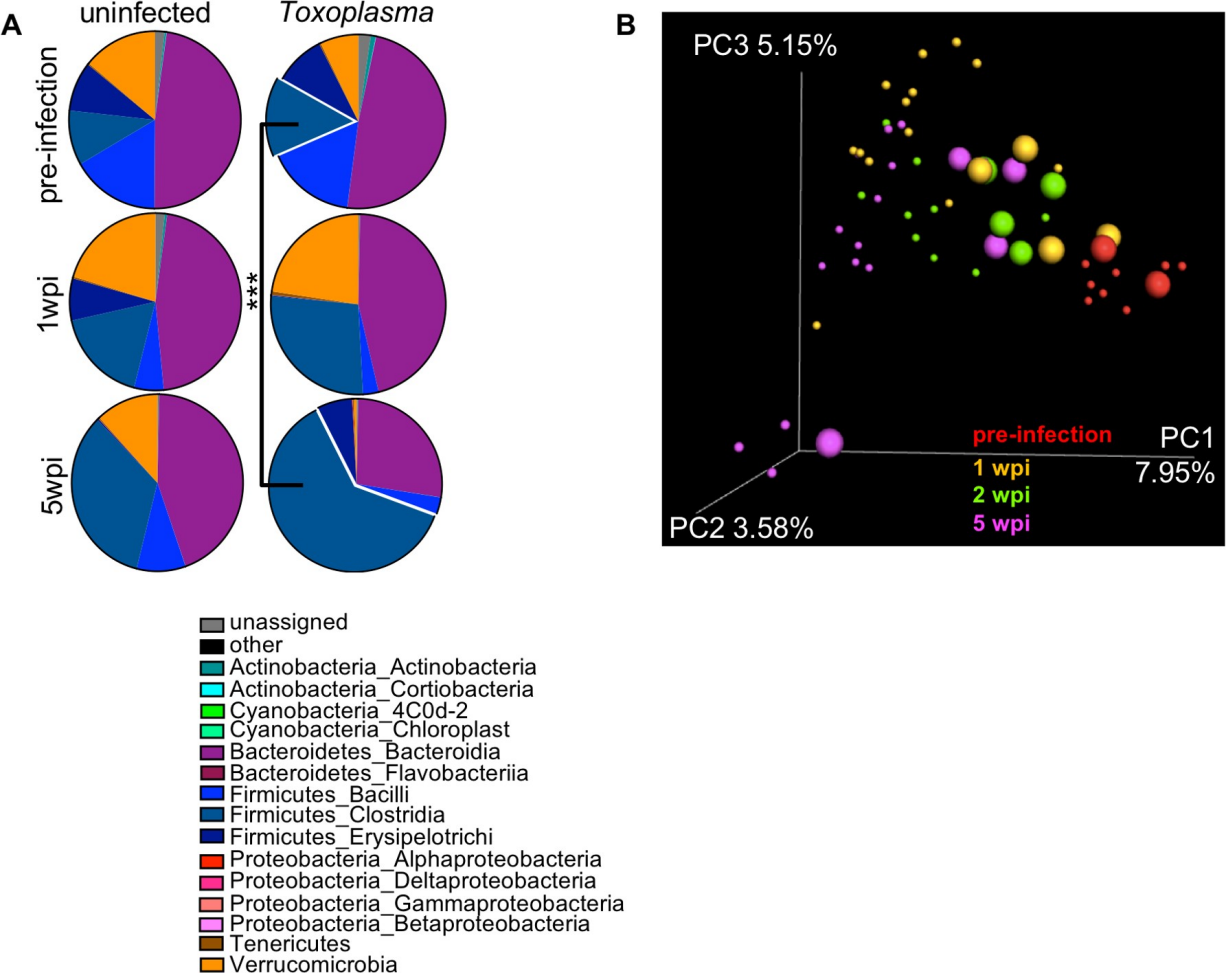
Changes in the commensal community are amplified in chronic infection. 16S profiling of commensal microbiota in fecal pellets gathered 2 days before infection (pre-infection), 1wpi or 5wpi with 120 *Toxoplasma* cysts. **A**, At chronic infection there is a significant outgrowth of *Firmicutes Clostridia* in comparison to pre-infection (navy blue, outset). *Toxoplasma* infected group: pre-infection mean 0.147±0.015 SEM, 5wpi mean 0.599±0.142 SEM, ***p≤0.001 student’s T-test. Data represented as the mean of 3–8 mice per time point. **B**, Unweighted Unifrac principle component analysis of 16S ribosomal subunit diversity in the fecal pellets of mice 2 days before infection (pre-infection, red), 1 wpi (orange), 2 wpi (green) or 5 wpi (magenta). Small circles = infected, large circles = uninfected. Data representative of two experiments.

Several groups have observed that acute infection with *Toxoplasma* triggers a loss of microbial diversity, an enrichment in Gram negative bacteria associated with intestinal pathology, and, sometimes, lethal ileitis[7,9,36]. However, it is not known if these changes in the commensal communities are transient or sustained in chronic infection. To address this, we collected fecal pellets from mice two days before infection (Fig 3 pre-infection) at 1 wpi or 5 wpi with *Toxoplasma* and analyzed microbial diversity by 16S ribosomal sequencing (Fig 3). In each cage, 1–2 uninfected animals were co-housed with infected littermate controls.

Community composition analysis reflected significant expansion in *Clostridia spp*. Operational Taxonomic Units (OTUs) 5 weeks post-*Toxoplasma* infection when compared to the pre-infection community (Fig 3A navy blue outset, 5 wpi 0.599±0.142 versus pre-infection 0.147±0.015 SEM, p = 0.0004, q = 0.007 student’s T-test). This trend was also observed in uninfected animals, although it was not statistically significant (5wpi 0.373±0.181 versus pre-infection 0.100±0.005 SEM, p = 0.319, q = 0.569). There was also a moderate, expansion in Verrucomicrobia at 1 week in both infected and uninfected animals that contracted by 5 weeks, although this change was not significant. The enrichment of *Clostridia spp*. in fecal pellets of infected mice was unexpected, based on previous observations that *Toxoplasma* infection can trigger an outgrowth of γ-proteobacteria in the lumen of the small intestine 7–9 dpi[7,8,29,36]. However, these mice were obtained from Jackson Lab (C57BL/6J) whereas previous reports describing γ-proteobacteria outgrowth were performed using C57BL/6N mice. In addition to harboring several important polymorphisms, C57BL/6N raised at the NIH are known to harbor an O21:H+ strains of *E*. *coli* associated with invasiveness (septicemia) and worse pathology in DSS colitis, AOFM-induced colon cancer and a range of gastro-intestinal bacterial infections[7,29,37,38]. In the context of these previous reports, our data are consistent with a model where the outgrowth species may reflect the facultative pathogens already present in the community (dependent on mouse genetic background and facility to facility variation) that capitalize on niche availability following an inflammatory insult. The outgrowth species, in turn is likely to have important implications on the immune response at both the site of the initial infection and systemically. These differences in the pre-existing members of the commensal community explain why our mice are more resistant to a high dose infection with Me49 cysts than others have reported in the past> As these pathological strains of e. coli have been associated with invasiveness, this may also explain why others have observed that Peyer’s patches become necrotic in acute *Toxoplasma* infection where we do not (Fig 2Ciii 20x and 2G 1wpi 40x)[9,21,35]. A second parameter that may explain why our mice are tolerant of high dose infection is that uninfected and infected animals were co-housed for the duration of our study. Coprophagia may have buffered the severity of the commensal shift in infected mice as well as altered the commensal population in uninfected animals, as discussed below.

When principal component analysis was used to assess beta diversity across fecal pellets, pre-infection animals (Fig 3B red) clustered distinctly from infected animals (Fig 3B small circles: yellow = 1 wpi, green = 2 wpi; magenta = 5 wpi). The shift in similarity away from pre-infected phenotype was most pronounced by 5 wpi. (Fig 3B magenta, small circles = infected, large circles = uninfected).Interestingly, the co-housed uninfected animals had a shift in microbial diversity as well and represented an intermediate cluster between pre-infection and infected animals (Fig 3B, 1 wpi yellow, large circles; 2 wpi green, large circles). As co-housed, uninfected animals do not display symptoms of cachexia we conclude that the observed changes to the microbial species are not sufficient to explain the cachexia phenotype alone. However, future studies will be needed to understand if the altered commensal community synergizes with immune or metabolic defects to promote cachexia maintenance.

## Conclusions

Here we describe a sustained cachexia phenotype in adult C57BL/6 mice (age 10–12 weeks) following per oral *Toxoplasma* infection. *Toxoplasma* cachexia is characterized by a loss of 20% in body mass, including fat and muscle, transient anorexia and an acute elevation in the hallmark cachexia cytokines IL-1, TNF and IL-6. To our knowledge, *Toxoplasma* infection is the first model to study sustained cachexia in mice that meets the modern, standard definition of cachexia put forth in 2008[2]. This will be an important tool for future studies aimed at understanding the physiological and molecular mechanism of cachexia. It may even prove to be a model to test interventions that halt and potential reverse disease.

*Toxoplasma* infection is well established to result in acute regional ileitis, however, a detailed analysis of how long intestinal inflammation is sustained or whether acute changes in commensal microbial communities are long lived has not been asked until now. Unexpectedly, we observed parasite signal in the same region of the distal jejunum throughout acute infection, suggesting that this is the major region of the small intestine supporting parasite replication. Perhaps more surprising, we found no evidence of sustained intestinal inflammation at 5wpi. In addition, the changes in fecal commensal communities observed in acute infection became more polarized in chronic infection rather than recovering as the intestinal barrier appeared to do. Importantly, the commensal communities of co-housed infected and uninfected mice both shifted by 5 weeks post inoculation. However, uninfected animals showed no signs of disease, suggesting that altered commensal microbiota alone is not sufficient to explain the sustained cachexia phenotype in infected animals.

Whether the cachexia program is beneficial to the host or the parasite remains to be determined. Anorexia and depletion of fat stores are classic signatures of infection that play an important role in restricting systemic bacterial pathogen replication but can trigger a host-detrimental response during viral infection[39,40]. In tissue culture, the *Toxoplasma* vacuole accumulates host lipids and parasite growth can be inhibited by blocking host lipases, suggesting that the lipolysis mobilized early in infection could benefit the parasite, although this has not been tested in vivo[41,42]. It is now well accepted that *Toxoplasma* infection triggers altered aversion behavior to feline urine[15,16]. This is hypothesized to be an adaptive strategy used by the parasite to facilitate transmission to the feline definitive host. Interestingly, the reduction in muscle mass during chronic *Toxoplasma* infection has been associated with reduced strength[22]. Therefore, it is plausible that promoting cachexia during chronic infection represents a second adaptive strategy used by *Toxoplasma* to facilitate the likelihood of predation by the definitive feline host. By studying the pathways that *Toxoplasma* has evolved to manipulate to promote transmission, we may identify critical immune and metabolic interactions driving the progression of chronic cachexia that can be applied to other disease settings.

## Supporting information

S1 Fig C57BL6/J and CBA/J mice are susceptible to cachexia following *Toxoplasma* infection however BALAB/c mice are not. BALB/c (orange square) or C57BL6/J mice (black square, B6 BALB cont.); CBA/J (blue circles) or C57BL6/J (black circles, CBA cont.) were infected with 120–200 cysts or mock infected. Weight was monitored at indicated time points. N = 4–8 mice per condition averaged across two independent experiments, significance is measured relative to uninfected at same time point.
(TIFF)
