# Supplementary figures and images for: Toxoplasma gondii infection triggers chronic cachexia and sustained commensal dysbiosis in mice
Source: PLoS One. 2018 Oct 31;13(10):e0204895. doi: 10.1371/journal.pone.0204895 (PMC6209157; doi:10.1371/journal.pone.0204895)

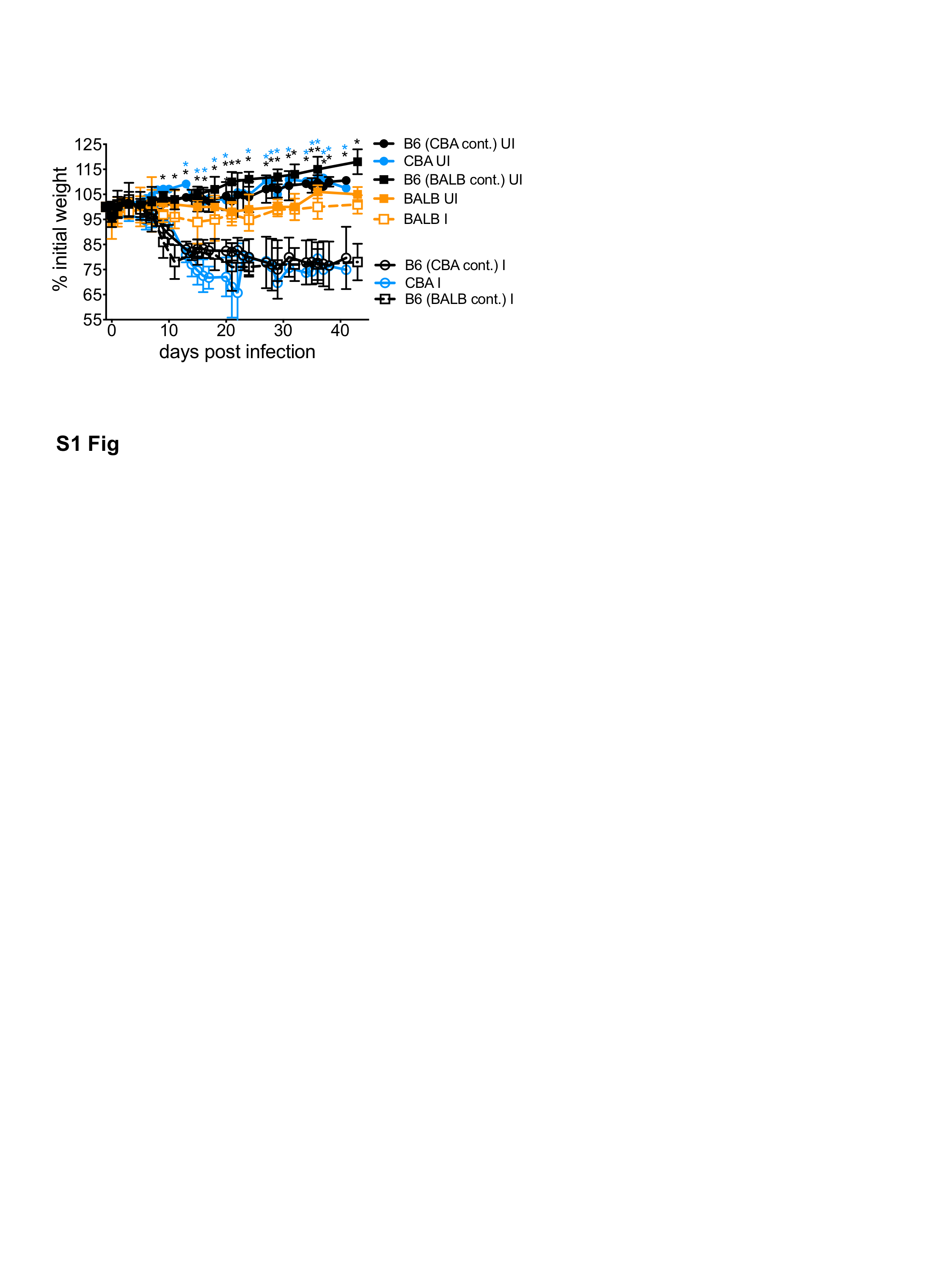

Supplement: S1 Fig — BALB/c (orange square) or C57BL6/J mice (black square, B6 BALB cont.); CBA/J (blue circles) or C57BL6/J (black circles, CBA cont.) were infected with 120–200 cysts or mock infected. Weight was monitored at indicated time points. N = 4–8 mice per condition averaged across two independent experiments, significance is measured relative to uninfected at same time point. (TIFF) [file pone.0204895.s001.tiff]
